# Overexpression of bovine leukemia virus receptor SLC7A1/CAT1 enhances cellular susceptibility to BLV infection on luminescence syncytium induction assay (LuSIA)

**DOI:** 10.1186/s12985-020-01324-y

**Published:** 2020-04-22

**Authors:** Hirotaka Sato, Lanlan Bai, Liushiqi Borjigin, Yoko Aida

**Affiliations:** 1 Nakamura Laboratory, Baton Zone Program, RIKEN Cluster for Science, Technology and Innovation Hub, 2-1 Hirosawa, Wako, Saitama, 351-0198 Japan; 2 Virus Infectious Diseases Unit, RIKEN, 2-1 Hirosawa, Wako, Saitama, 351-0198 Japan; 3 Photonics Control Technology Team, RIKEN Center for Advanced Photonics, 2-1 Hirosawa, Wako, Saitama, 351-0198 Japan

**Keywords:** Bovine leukemia virus, Receptor, CAT1/SLC7A1, Luminescence syncytium induction assay, Infection

## Abstract

Bovine leukemia virus (BLV) causes enzootic bovine leukosis, the most common neoplastic disease in cattle. We previously reported the development and protocol of the luminescence syncytium induction assay (LuSIA), a method for evaluating BLV infectivity based on CC81-GREMG cells. These cells form syncytia expressing enhanced green fluorescent protein when co-cultured with BLV-infected cells. Recently, we confirmed CAT1/SLC7A1 functions as a receptor of BLV. Here, we focused on CAT1/SLC7A1 to increase the sensitivity of LuSIA. We constructed a bovine CAT1-expressing plasmid and established a new CC81-GREMG-derived reporter cell line highly expressing bovine CAT1 (CC81-GREMG-CAT1). The new LuSIA protocol using CC81-GREMG-CAT1 cells measures cell-to-cell infectivity and cell-free infectivity of BLV faster and with greater sensitivity than the previous protocol using CC81-GREMG. The new LuSIA protocol is quantitative and more sensitive than the previous assay based on CC81-GREMG cells and will facilitate the development of several new BLV assays.

## Main text

Bovine leukemia virus (BLV), the causative agent of enzootic bovine leukosis, and, a B-cell leukemia/lymphoma, belongs to family *Retroviridae*. BLV is an oncogenic member of the genus *Deltaretrovirus*, which also includes human T-lymphotropic virus types 1 and 2 [1]. Currently, BLV is widely distributed in cattle populations [2–7], causing serious problems in the cattle industry without the onset of leukosis. For example, BLV infection decreases milk production, carcass weight and cow lifespan [8].

BLV is mainly transmitted via cell-containing fluids such as blood and milk by cell-to-cell contact. Although BLV can infect CD4^+^T cells, CD8^+^T cells, γ/T-cells, monocytes, and granulocytes in cattle [9–16], many tumor cells are derived from CD5^+^IgM^+^B-cell subpopulations [12, 17]. In addition to the many studies showing that the BLV host range is broad, BLV can successfully infect a variety of cells in vitro [18]. We confirmed that cationic amino acid transporter 1 (CAT1/SLC7A1: CAT1), which is ubiquitously expressed on cells in the whole body, functions as a receptor of BLV infection and is responsible for the broad host range of BLV in vitro [19]*.* CAT1 has 14 potential membrane-spanning domains, is ubiquitously expressed in a wide variety of cultured cell lines, and plays essential roles in basic cellular function [20].

BLV infectivity is typically measured by the syncytium induction assay (SIA) [21, 22]. We previously developed a method for visualizing BLV infectivity known as the luminescence syncytium induction assay (LuSIA), which uses CC81-BLU3G as a reporter cell line [23]. CC81-BLU3G cells are stably transfected with a pBLU3-EGFP reporter plasmid harboring the BLV- long terminal repeat (LTR) U3 region as the promoter and enhanced green fluorescent protein (EGFP) as the reporter gene. When CC81-BLU3G cells are infected with BLV, they form large multinuclear syncytia that express EGFP. To improve sensitivity and reduce background fluorescence, we developed a more sensitive LuSIA using a 2nd generation reporter cell line, CC81-GREMG (GREMG; glucocorticoid response element mutated reporter cording with EGFP), which was stably transfected with a reporter plasmid bearing a mutated glucocorticoid response element on the LTR U3 region promoter [24].

CAT1 protein appears to function as a cell surface receptor for BLV infection [19]. Therefore, CAT1 expression on each cell is correlated with individual cellular susceptibility to BLV infection. In the current study, we predicted that a new LuSIA based on a new reporter cell line overexpressing CAT1 protein would be more susceptible to BLV infectivity compared to the present protocol of LuSIA based on parent CC81-GREMG. We first developed a new reporter cell line, CC81-GREMG-CAT1, which showed higher expression compared to the parent CC81-GREMG cells by stable transfection of the bovine CAT1/SLC7A1 expression plasmid and then constructed a 3rd generation LuSIA based on CC81-GREMG-CAT1. We then compared the sensitivity of the assay to cell-free infection and cell-to-cell infection evaluated by the 2nd generation LuSIA based on CC81-GREMG.

**Fig. 1:**
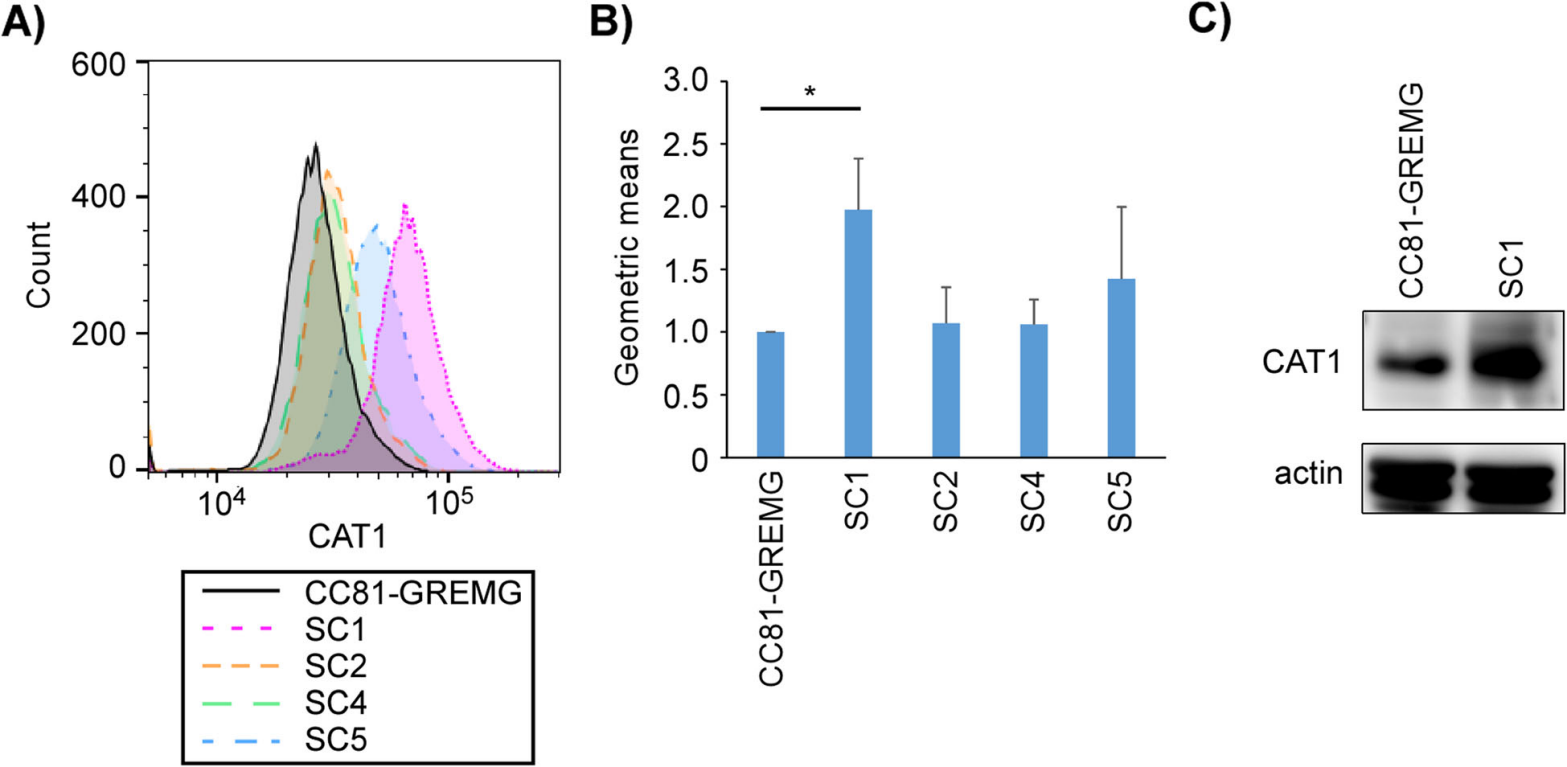
CAT1 protein expression on CC81-GREMG cells and newly established bovine CAT1 stably transfected reporter cell clones. **a** CAT1 expression histogram of flow cytometric analysis. Four CAT1 stably transfected clones (SC1, SC2, SC4 and SC5) were fixed with 1% formaldehyde/PBS and permeabilized with 0.1% TritonX-100/PBS prior to staining with rabbit anti-CAT1 polyclonal antibody (Abcam, Cambridge, UK) and AlexaFluor 647 goat anti-rabbit antibody. Permeabilization allowed antibody binding to an intracellular region of CAT1 protein. CAT1 protein expression was measured with a BD Accuri C6sampler plus (BD Biosciences, San Jose, CA, USA) and analyzed by FlowJo software Ver.10 (FlowJo, LLC, Ashland, OR, USA). **b** Geometric mean expression of CAT1 in A. Mean and standard deviation of three independent experiments. The asterisk (*) represents a *p*-value of 0.05. **c** Western blot analysis of CC81-GREMG and CAT1 stably transfected single clone SC1 cell with anti-CAT1 (upper panel) and anti-β-actin antibodies (bottom panel). CC81-GREMG and SC1 cells were lysed with lysis buffer (50 mM Tris-HCl, pH 8.0, 150 mM sodium chloride, 0.5% sodium deoxycholate, 0.1% sodium dodecyl sulfate (SDS), 1.0% Nonidet P-40) and mixed with 4x SDS-sample buffer. Ten micrograms of total protein were applied to 10% SDS-polyacrylamide gel and transferred to a polyvinylidene difluoride membrane. CAT1 protein was detected by rabbit anti-CAT1 polyclonal antibody, horseradish peroxidase-conjugated rat anti-rabbit IgG antibody (Amersham Biosciences, Piscataway, NJ, USA), and SuperSignal™ West Pico Chemiluminescent Substrate (Thermo Fisher Scientific)

To construct the bovine CAT1-expressing plasmid, RNAs were extracted from the bovine lymphoid cell line KU1 and then reverse-transcribed into cDNA using a high- capacity RNA-to-cDNA kit (Thermo Fisher Scientific, Waltham, MA, USA). CAT1 was amplified by PCR using PrimeSTAR GXL (Takara Bio, Shiga, Japan) and digested by *EcoR*I and *Not*I restriction enzymes and ligated into the pME18neo expression vector. The neomycin resistance gene was recombined with the hygromycin resistance gene using an In-Fusion HD cloning kit (TaKaRa Bio). The constructed plasmid was designated as pME-CAT1hyg and stably transfected into CC81-GREMG cell using Lipofectamine 3000 regent (Thermo Fisher Scientific). Transfected cells were cultured in Dulbecco’s modified Eagle’s Medium (Thermo Fisher Scientific) supplemented with 10% fetal bovine serum (Sigma-Aldrich, St. Louis, MO, USA), 250 μg/mL hygromycin B (Sigma-Aldrich), and 500 μg/mL G-418 (Roche, Basel, Switzerland) for several weeks. Single clones were selected by limited dilution in a 96-well culture plate until growth. Finally, four CAT1 stably transfected clones were established. To compare the expression level of CAT1 protein in the four clones, CAT1 expression was evaluated by flow cytometry with rabbit anti-CAT1 polyclonal antibody, which binds to an intracellular region of CAT1 protein followed by treatment with AlexaFluor 647 goat anti-rabbit antibody (Thermo Fisher Scientific). All CAT1 stably transfected clones showed higher expression of CAT1 protein than the parental cell line CC81-GREMG (Fig. 1a and b). Particularly, clone SC1 showed the significantly highest expression of CAT1 protein among the four clones. To confirm this result, CAT1 expression in CC81-GREMG and SC1 cells was measured by western blot analysis with an anti-CAT1 antibody (Fig. 1c). The SC1 clone highly expressed CAT1 protein compared to CC81-GREMG cells. Therefore, we selected the SC1 clone as reporter cell line which was designated as CC81-GREMG-CAT1.

**Fig. 2:**
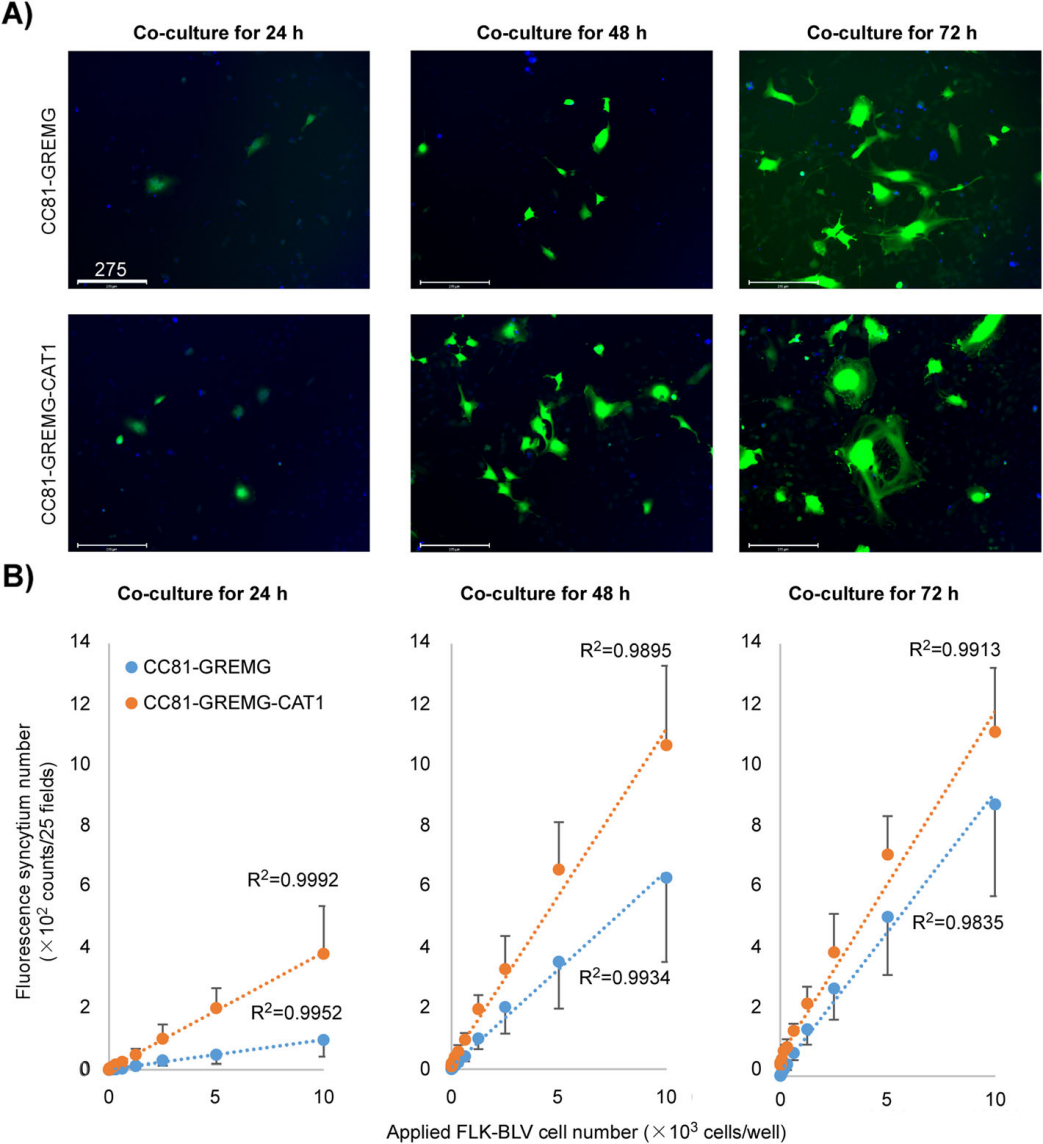
Comparison of quantitative analysis of cell-to-cell infection by LuSIAs using CC81-GREMG-CAT1 and CC81-GREMG. **a** CC81-GREMG-CAT1 or CC81-GREMG cells (2 × 10^5^ cells/well) were cultured with or without 5000 cells/well of FLK-BLV cells, which are productively infected by BLV at 37 °C for 24, 48, and 72 h. Fluorescent syncytia were detected with an EVOS2 fluorescence microscope (Thermo Fisher Scientific) and analyzed with HCS studio software (Thermo Fisher Scientific). The scale bar (white bars) indicates 275 μm. **b** Correlation of the number of EGFP-expressing syncytia and FLK-BLV cell number, when CC81-GREMG-CAT1 or CC81-GREMG cells were cultured with serially diluted FLK-BLV cells (10,000, 5000, 2500, 1250, 625, 312, 156, 78, 39, 20, 10, 0 cells/well) for 24 and 72 h. The results indicate the mean and standard deviation of three independent experiments

Next, we evaluated the sensitivity of the 3rd generation LuSIA based on CC81-GREMG-CAT1. To compare the sensitivity of the newly developed reporter cell line CC81-GREMG-CAT1 and its parental reporter cell line CC81-GREMG, the cells were cultured with 5000 cells of FLK-BLV (a fetal lamb kidney cell line constitutively expressing BLV), which were infected by BLV for 24, 48, and 72 h. Fluorescent syncytia were detected with an EVOS2 fluorescence microscope and analyzed with HCS studio software (Fig. 2a). At 24 h post-cultivation, in LuSIAs using both reporter cells, CC81-GREMG-CAT1 (202.3 ± 65.8 counts/well) formed a larger number of fluorescing syncytia than CC81-GREMG (48.7 ± 29.9 counts/well). Additionally, fluorescing syncytia by CC81-GREMG-CAT1 were larger than those formed by CC81-GREMG. The same tendencies were observed at 24, 48, and 72 h post- cultivation (Fig. 2a). Furthermore, to evaluate the sensitivity of CC81-GREMG-CAT1, both reporter cells were cultured with serially diluted FLK-BLV cells for 24, 48 and 72 h. In LuSIAs using both reporter cell lines, the number of fluorescent syncytia was strongly correlated with the number of FLK-BLV cells (R^2^ = 0.999 for CC81-GREMG-CAT1; R^2^ = 0.995 for CC81-BLU3G) at 24 h post-cultivation (Fig. 2b). The numbers of fluorescent syncytia obtained by LuSIA were higher when using CC81-GREMG-CAT1 (381.7 ± 157.1 to 2.3 ± 1.5 counts/well) than when using CC81-GREMG (97.3 ± 55.4 to 0.7 ± 0.6 counts/well) at 24 h post-cultivation. The same tendencies were observed at 48 and 72 h post-cultivation (Fig. 2b). We previously reported that 2nd generation LuSIA using CC81-GREMG was more sensitive than the 1st generation LuSIA using CC81-BLU3G [24]. Our results demonstrated that CC81-GREMG-CAT1 is more suitable for analyzing cell-to-cell infectivity of BLV by LuSIA.

**Fig. 3:**
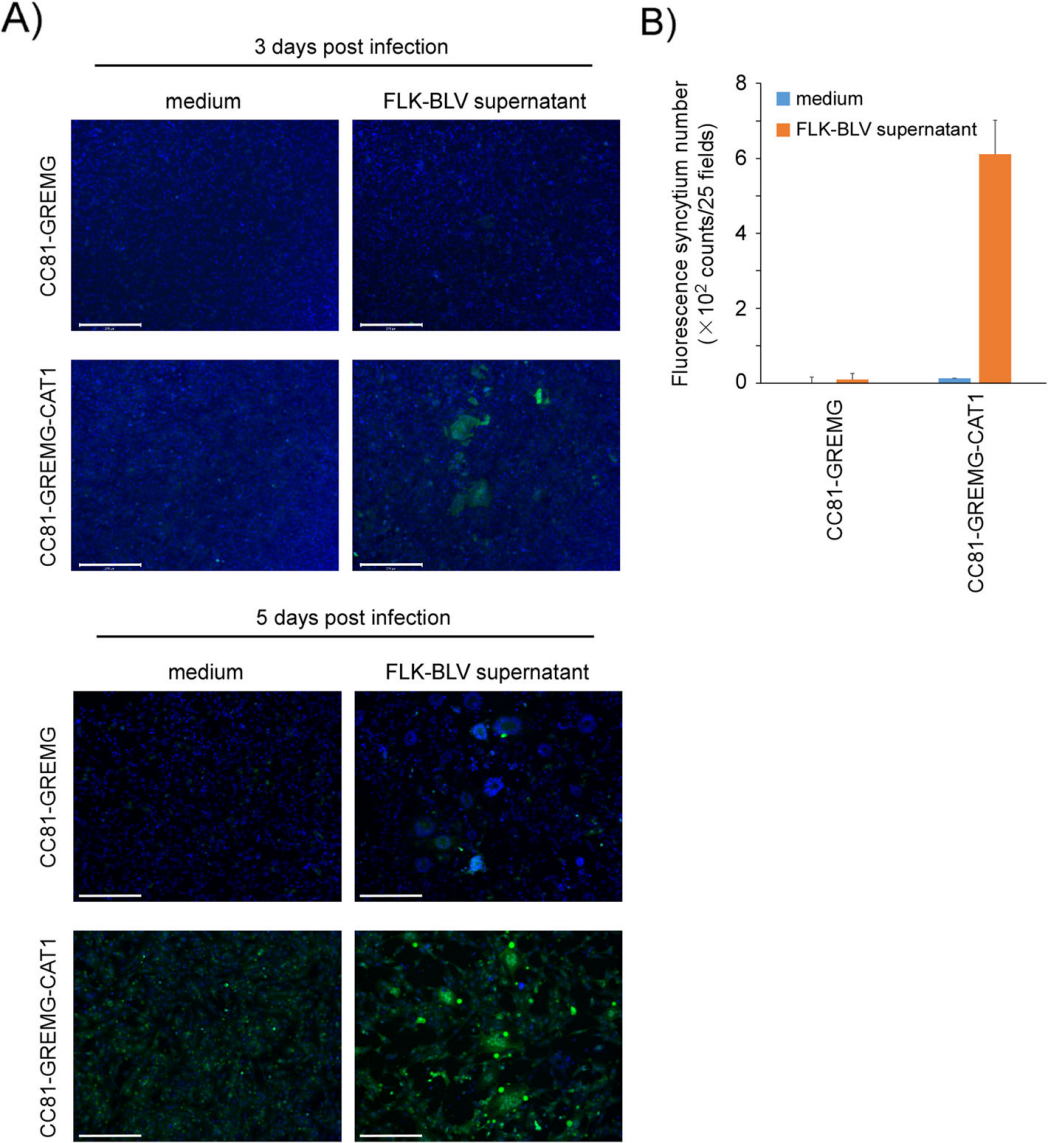
Comparison of detection of cell-free infection by LuSIAs using CC81-GREMG-CAT1 and CC81-GREMG. **a**) CC81-GREMG-CAT1 and CC81-GREMG cells (4 × 10^5^ cells/well) were cultured with or without culture supernatant (containing 94 ng/well of BLV p24 protein) collected from FLK-BLV cells. The cells were cultured including 20 ng/mL of Hoechst 33342 (Sigma-Aldrich) and fluorescent syncytia are observed daily with an EVOS2 fluorescence microscope and analyzed with HCS studio software. The scale bar (white bars) indicate 275 μm. **b** Fluorescent syncytia were detected by LuSIAs using CC81-GREMG-CAT1 and CC81-GREMG at 3 days post-cultivation. The results indicate the mean and standard deviation of three independent experiments

Previously, we reported that cell-free infection of the FLK-BLV supernatant was detectable by LuSIA using CC81-GREMG after 5 days of cultivation [24]. Here, we attempted to use CC81-GREMG-CAT1 cells to detect cell-free infection of BLV by decreasing the co-culture time to 3 days from 5 days using 2nd generation LuSIA based on CC81-GREMG. CC81-GREMG-CAT1 and CC81-GREMG were cultured with 20 ng/mL of Hoechst 33342 and infected with culture supernatant collected from FLK-BLV cells. The fluorescent syncytia were clearly detected in CC81-GREMG-CAT1 at 3 days post-cultivation, whereas fluorescent syncytia were detected in CC81-GREMG at 5 days post-cultivation (Fig. 3a and b), indicating that CC81-GREMG-CAT1 shows more rapid results with higher sensitivity for BLV cell-free infection than CC81-GREMG. This result indicates that the 3rd generation LuSIA based on CC81-GREMG-CAT1 can detect infectious BLV particles within 3 days, which was not detected by 2nd generation LuSIA.

Our result confirmed the hypothesis that overexpression of the BLV receptor bovine CAT1 enhances the cellular susceptibility to BLV infection, thereby increasing the sensitivity of LuSIA. (i) CC81-GREMG-CAT1 can detect cell-free infection of BLV at an earlier time point by decreasing the co-culture time to 3 days from 5 days using in 2nd generation LuSIA based on CC81-GREMG. (ii) CC81-GREMG-CAT1 is more suitable for analyzing the cell-to-cell infectivity of BLV by LuSIA, as CC81-GREMG-CAT1 formed a larger number and bigger fluorescing syncytium than CC81-GREMG. Thus, the new LuSIA protocol using the 3rd generation reporter cell line CC81-GREMG-CAT1, which showed higher expression of the BLV receptor CAT1, is advantageous for earlier and sensitive detection of both cell-to-cell and cell-free infection of BLV (Table 1.). Additionally, this assay can be further developed to visualize the infectivity of BLV in a more sensitive and/or rapid manner, such as in a BLV contamination contradiction assay of bovine vaccines.

**Table 1:** Development of luminescence syncytium induction assay

methods	reporter cell lines	identification	Non-specific background	BLV-infected cells	BLV particle	approved for milk sample	references
SIA	CC81, F81	Giemsa staining	high	+	+	–	[21, 22]
LuSIA 1st. Gen.	CC81-BLU3G	auto-fluorescence	high	++	++	–	[23]
LuSIA 2nd. Gen.	CC81-BLU3L	luciferase assay	low	++	++	NT	[24]
LuSIA 2nd. Gen.	CC81-GREMG	auto-fluorescence	low	++	++	+	[24, 25]
LuSIA 3rd. Gen.	CC81-GREMG-CAT1	auto-fluorescence	medium	+++	+++	NT	this report

## Data Availability

All data analyzed for the purposes of this manuscript are included in this article.

## Abbreviations

BLV: Bovine leukemia virus
CAT1: cationic amino acid transporter 1
EGFP: enhanced green fluorescent protein
FLK: fetal lamb kidney
GREMG: glucocorticoid response element mutated reporter cording with EGFP
LTR: long terminal repeat
LuSIA: luminescence syncytium induction assay
PBS: phosphate-buffered saline
SDS: sodium dodecyl sulfate
SIA: syncytium induction assay
